# *N*-(4-Fluoro­phen­yl)-2,6-dimethyl-1,3-dioxan-4-amine

**DOI:** 10.1107/S1600536813026561

**Published:** 2013-10-02

**Authors:** Gottimukkala Rambabu, Zeenat Fatima, Bandapalli Palakshi Reddy, Vijayaparthasarathi Vijayakumar, Devadasan Velmurugan

**Affiliations:** a Chemistry Department, GEBH, Sree Vidyanikethan Engineering College, A. Rangampet, Tirupati 517 102, India; b Centre of Advanced Study in Crystallography and Biophysics, University of Madras, Guindy Campus, Chennai 600 025, India; c Centre for Organic and Medicinal Chemistry, VIT University, Vellore 632 014, India

## Abstract

In the title compound, C_12_H_16_FNO_2_, the dioxane ring adopts a chair conformation with the methyl substituents and the C—N bond in equatorial orientations. Its mean plane subtends a dihedral angle of 40.17 (6)° with the benzene ring. In the crystal, weak N—H⋯F hydrogen bonds link the mol­ecules into *C*(7) chains propagating in [100].

## Related literature

For a related structure and background to dioxanes, see: Fatima *et al.* (2013 ▶).
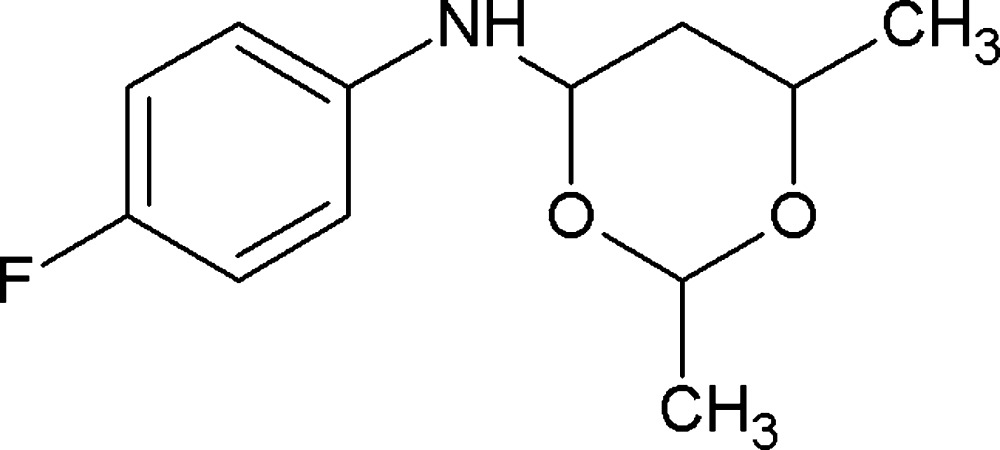


## Experimental

### 

#### Crystal data

C_12_H_16_FNO_2_
*M* *_r_* = 225.26
Monoclinic, 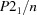

*a* = 10.4924 (10) Å
*b* = 10.0614 (10) Å
*c* = 11.0379 (11) Å
β = 90.136 (2)°
*V* = 1165.2 (2) Å^3^
*Z* = 4
Mo *K*α radiation
μ = 0.10 mm^−1^
*T* = 293 K
0.25 × 0.20 × 0.15 mm

#### Data collection

Bruker SMART APEXII area-detector diffractometer
Absorption correction: multi-scan (*SADABS*; Bruker, 2008 ▶) *T*_min_ = 0.649, *T*_max_ = 0.746
10977 measured reflections
2910 independent reflections
2179 reflections with *I* > 2σ(*I*)
*R*_int_ = 0.020

#### Refinement

*R*[*F* ^2^ > 2σ(*F* ^2^)] = 0.041
*wR*(*F* ^2^) = 0.129
*S* = 1.01
2910 reflections
147 parameters
H-atom parameters constrained
Δρ_max_ = 0.15 e Å^−3^
Δρ_min_ = −0.18 e Å^−3^

### 

Data collection: *APEX2* (Bruker, 2008 ▶); cell refinement: *SAINT* (Bruker, 2008 ▶); data reduction: *SAINT*; program(s) used to solve structure: *SHELXS97* (Sheldrick, 2008 ▶); program(s) used to refine structure: *SHELXL97* (Sheldrick, 2008 ▶); molecular graphics: *ORTEP-3 for Windows* (Farrugia, 2012 ▶); software used to prepare material for publication: *SHELXL97* and *PLATON* (Spek, 2009 ▶).

## Supplementary Material

Crystal structure: contains datablock(s) global, I. DOI: 10.1107/S1600536813026561/hb7138sup1.cif

Structure factors: contains datablock(s) I. DOI: 10.1107/S1600536813026561/hb7138Isup2.hkl

Supplementary material file. DOI: 10.1107/S1600536813026561/hb7138Isup3.cml

Additional supplementary materials:  crystallographic information; 3D view; checkCIF report

## Figures and Tables

**Table 1:** Hydrogen-bond geometry (Å, °)

*D*—H⋯*A*	*D*—H	H⋯*A*	*D*⋯*A*	*D*—H⋯*A*
N1—H1⋯F1^i^	0.86	2.48	3.1556 (14)	136

Symmetry code: (i) 
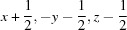
.

**Fig. 1.**
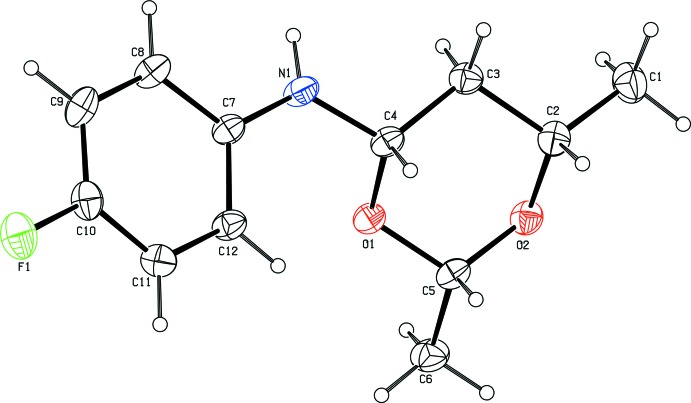
The molecular structure of the title compound, showing displacement ellipsoids drawn at the 30% probability level.

**Fig. 2.**
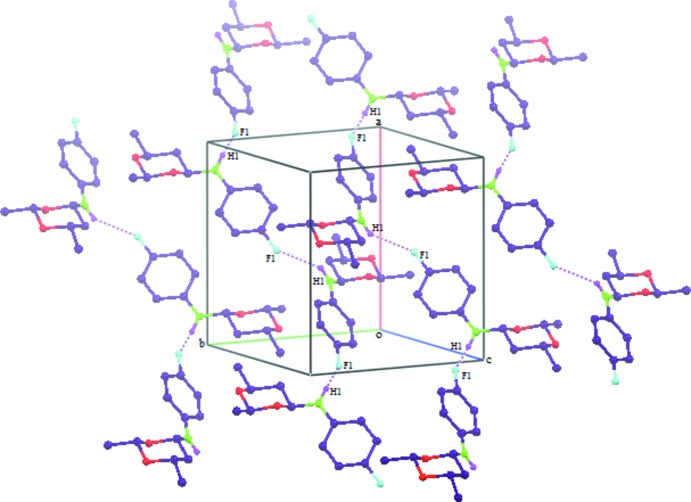
The crystal packing of the title compound viewed down a axis. H-atoms not involved in H-bonds have been excluded for clarity.

## supplementary crystallographic information

### 1. Comment

As part of our ongoing studies of dioxane derivatives with possible biological activity (Fatima *et al.*, 2013), we now describe the single-crystal structure determination of the title compound.

Molecules of the title compound, C_12_H_16_N_1_O_2_F_1_, (Fig.1) are linked by intermolecular N—H···F hydrogen bonds into infinite chains propagating along 'a' axis (Fig. 2). The dioxane ring (O1/O2/C2—C5) adopts a *chair* conformation and the best plane through the dioxane ring makes a dihedral angle of 40.17 (6)° with the phenyl ring (C7—C12).

### 2. Experimental

To 4-Fluoroaniline (1 mmol), Acetaldehyde (3 mmol) was added dropwise and stirred for about 4 h at 0 °C. The progress of the reaction was monitored through TLC. The reaction mixture was washed with petroleum ether. Resultant was dissolved in diethylether and allowed to evaporate. Solid product obtained was recrystallized with diethylether.

### 3. Refinement

The hydrogen atoms were placed in calculated positions with C—H = 0.93 Å to 0.98 Å refined in the riding model with fixed isotropic displacement parameters:*U*_iso_(H) = 1.5*U*_eq_(C) for methyl group and *U*_iso_(H) = 1.2*U*_eq_(C) for other groups.

### Crystal data

**Table:**

C_12_H_16_FNO_2_	*F*(000) = 480
*M**_r_* = 225.26	*D*_x_ = 1.284 Mg m^−^^3^
Monoclinic, *P*2_1_/*n*	Mo *K*α radiation, λ = 0.71073 Å
Hall symbol: -P 2yn	Cell parameters from 2910 reflections
*a* = 10.4924 (10) Å	θ = 2.7–28.4°
*b* = 10.0614 (10) Å	µ = 0.10 mm^−^^1^
*c* = 11.0379 (11) Å	*T* = 293 K
β = 90.136 (2)°	Block, colourless
*V* = 1165.2 (2) Å^3^	0.25 × 0.20 × 0.15 mm
*Z* = 4	

### Data collection

**Table:**

Bruker SMART APEXII area-detector diffractometer	2910 independent reflections
Radiation source: fine-focus sealed tube	2179 reflections with *I* > 2σ(*I*)
Graphite monochromator	*R*_int_ = 0.020
ω and φ scans	θ_max_ = 28.4°, θ_min_ = 2.7°
Absorption correction: multi-scan (*SADABS*; Bruker, 2008)	*h* = −13→13
*T*_min_ = 0.649, *T*_max_ = 0.746	*k* = −13→13
10977 measured reflections	*l* = −14→14

### Refinement

**Table:**

Refinement on *F*^2^	Primary atom site location: structure-invariant direct methods
Least-squares matrix: full	Secondary atom site location: difference Fourier map
*R*[*F*^2^ > 2σ(*F*^2^)] = 0.041	Hydrogen site location: inferred from neighbouring sites
*wR*(*F*^2^) = 0.129	H-atom parameters constrained
*S* = 1.01	*w* = 1/[σ^2^(*F*_o_^2^) + (0.0644*P*)^2^ + 0.1851*P*] where *P* = (*F*_o_^2^ + 2*F*_c_^2^)/3
2910 reflections	(Δ/σ)_max_ < 0.001
147 parameters	Δρ_max_ = 0.15 e Å^−^^3^
0 restraints	Δρ_min_ = −0.18 e Å^−^^3^

### Special details

**Table:**

Geometry. All e.s.d.'s (except the e.s.d. in the dihedral angle between two l.s. planes) are estimated using the full covariance matrix. The cell e.s.d.'s are taken into account individually in the estimation of e.s.d.'s in distances, angles and torsion angles; correlations between e.s.d.'s in cell parameters are only used when they are defined by crystal symmetry. An approximate (isotropic) treatment of cell e.s.d.'s is used for estimating e.s.d.'s involving l.s. planes.
Refinement. Refinement of *F*^2^ against ALL reflections. The weighted *R*-factor *wR* and goodness of fit *S* are based on *F*^2^, conventional *R*-factors *R* are based on *F*, with *F* set to zero for negative *F*^2^. The threshold expression of *F*^2^ > σ(*F*^2^) is used only for calculating *R*-factors(gt) *etc*. and is not relevant to the choice of reflections for refinement. *R*-factors based on *F*^2^ are statistically about twice as large as those based on *F*, and *R*- factors based on ALL data will be even larger.

### Fractional atomic coordinates and isotropic or equivalent isotropic displacement parameters (Å^2^)

**Table:**

	*x*	*y*	*z*	*U*_iso_*/*U*_eq_	
O1	0.85420 (8)	0.16432 (8)	0.95248 (8)	0.0469 (2)	
O2	0.91002 (9)	0.28191 (9)	0.77911 (8)	0.0509 (2)	
C3	0.91619 (12)	0.04131 (13)	0.77606 (11)	0.0491 (3)	
H3A	0.8970	−0.0356	0.7263	0.059*	
H3B	1.0045	0.0351	0.8018	0.059*	
C5	0.82700 (12)	0.27629 (12)	0.87937 (12)	0.0482 (3)	
H5	0.7386	0.2718	0.8509	0.058*	
N1	0.84979 (10)	−0.06797 (11)	0.96310 (10)	0.0525 (3)	
H1	0.9177	−0.1148	0.9546	0.063*	
C4	0.83024 (11)	0.04228 (11)	0.88605 (11)	0.0453 (3)	
H4	0.7413	0.0421	0.8587	0.054*	
C2	0.89697 (13)	0.16759 (13)	0.70224 (12)	0.0514 (3)	
H2	0.8111	0.1669	0.6672	0.062*	
F1	0.49573 (9)	−0.21970 (11)	1.30521 (8)	0.0788 (3)	
C7	0.76261 (11)	−0.10329 (11)	1.05237 (11)	0.0442 (3)	
C8	0.77421 (13)	−0.22492 (13)	1.11186 (14)	0.0556 (3)	
H8	0.8424	−0.2806	1.0939	0.067*	
C9	0.68551 (15)	−0.26374 (15)	1.19728 (14)	0.0622 (4)	
H9	0.6935	−0.3450	1.2367	0.075*	
C12	0.66199 (11)	−0.02028 (12)	1.08440 (12)	0.0497 (3)	
H12	0.6546	0.0628	1.0481	0.060*	
C11	0.57272 (13)	−0.05951 (14)	1.16951 (12)	0.0539 (3)	
H11	0.5051	−0.0041	1.1898	0.065*	
C6	0.84563 (15)	0.39947 (14)	0.95358 (14)	0.0630 (4)	
H6A	0.9318	0.4026	0.9830	0.095*	
H6B	0.7879	0.3985	1.0209	0.095*	
H6C	0.8292	0.4763	0.9044	0.095*	
C10	0.58580 (13)	−0.18076 (15)	1.22298 (12)	0.0553 (3)	
C1	0.99285 (17)	0.18267 (17)	0.60184 (14)	0.0693 (4)	
H1A	0.9765	0.2637	0.5585	0.104*	
H1B	0.9860	0.1086	0.5474	0.104*	
H1C	1.0772	0.1854	0.6357	0.104*	

### Atomic displacement parameters (Å^2^)

**Table:**

	*U*^11^	*U*^22^	*U*^33^	*U*^12^	*U*^13^	*U*^23^
O1	0.0494 (5)	0.0386 (4)	0.0529 (5)	−0.0015 (3)	−0.0005 (4)	0.0021 (3)
O2	0.0567 (5)	0.0407 (5)	0.0551 (5)	−0.0081 (4)	−0.0008 (4)	0.0033 (4)
C3	0.0510 (7)	0.0415 (6)	0.0550 (7)	−0.0041 (5)	0.0010 (5)	−0.0027 (5)
C5	0.0448 (6)	0.0382 (6)	0.0615 (7)	0.0007 (5)	−0.0030 (5)	0.0043 (5)
N1	0.0438 (5)	0.0426 (5)	0.0712 (7)	0.0076 (4)	0.0087 (5)	0.0123 (5)
C4	0.0402 (6)	0.0372 (6)	0.0584 (7)	−0.0023 (4)	−0.0015 (5)	0.0035 (5)
C2	0.0558 (7)	0.0469 (7)	0.0514 (7)	−0.0082 (5)	−0.0057 (6)	0.0004 (5)
F1	0.0799 (6)	0.0875 (7)	0.0691 (6)	−0.0231 (5)	0.0156 (5)	0.0142 (5)
C7	0.0412 (6)	0.0360 (6)	0.0555 (7)	−0.0029 (4)	−0.0031 (5)	0.0024 (5)
C8	0.0480 (7)	0.0442 (7)	0.0747 (9)	0.0025 (5)	−0.0051 (6)	0.0114 (6)
C9	0.0643 (8)	0.0516 (7)	0.0705 (9)	−0.0076 (6)	−0.0097 (7)	0.0214 (7)
C12	0.0499 (6)	0.0372 (6)	0.0621 (7)	0.0001 (5)	0.0030 (5)	0.0039 (5)
C11	0.0503 (7)	0.0510 (7)	0.0603 (8)	−0.0029 (5)	0.0048 (6)	−0.0042 (6)
C6	0.0729 (9)	0.0416 (7)	0.0746 (9)	−0.0004 (6)	0.0029 (7)	−0.0027 (6)
C10	0.0554 (7)	0.0602 (8)	0.0502 (7)	−0.0158 (6)	0.0003 (6)	0.0050 (6)
C1	0.0872 (11)	0.0654 (9)	0.0554 (8)	−0.0120 (8)	0.0075 (8)	0.0026 (7)

### Geometric parameters (Å, º)

**Table:**

O1—C5	1.4145 (14)	C7—C8	1.3940 (17)
O1—C4	1.4517 (14)	C7—C12	1.3924 (17)
O2—C5	1.4111 (16)	C8—C9	1.383 (2)
O2—C2	1.4357 (15)	C8—H8	0.9300
C3—C4	1.5141 (17)	C9—C10	1.369 (2)
C3—C2	1.5226 (18)	C9—H9	0.9300
C3—H3A	0.9700	C12—C11	1.3856 (17)
C3—H3B	0.9700	C12—H12	0.9300
C5—C6	1.4981 (19)	C11—C10	1.362 (2)
C5—H5	0.9800	C11—H11	0.9300
N1—C7	1.3922 (15)	C6—H6A	0.9600
N1—C4	1.4125 (15)	C6—H6B	0.9600
N1—H1	0.8600	C6—H6C	0.9600
C4—H4	0.9800	C1—H1A	0.9600
C2—C1	1.5061 (19)	C1—H1B	0.9600
C2—H2	0.9800	C1—H1C	0.9600
F1—C10	1.3691 (15)		
			
C5—O1—C4	110.55 (9)	C8—C7—C12	118.19 (12)
C5—O2—C2	111.92 (9)	N1—C7—C12	121.77 (11)
C4—C3—C2	110.20 (10)	C9—C8—C7	120.73 (13)
C4—C3—H3A	109.6	C9—C8—H8	119.6
C2—C3—H3A	109.6	C7—C8—H8	119.6
C4—C3—H3B	109.6	C10—C9—C8	119.02 (13)
C2—C3—H3B	109.6	C10—C9—H9	120.5
H3A—C3—H3B	108.1	C8—C9—H9	120.5
O2—C5—O1	110.80 (9)	C11—C12—C7	121.03 (12)
O2—C5—C6	108.40 (10)	C11—C12—H12	119.5
O1—C5—C6	108.73 (11)	C7—C12—H12	119.5
O2—C5—H5	109.6	C10—C11—C12	118.78 (13)
O1—C5—H5	109.6	C10—C11—H11	120.6
C6—C5—H5	109.6	C12—C11—H11	120.6
C7—N1—C4	122.13 (10)	C5—C6—H6A	109.5
C7—N1—H1	118.9	C5—C6—H6B	109.5
C4—N1—H1	118.9	H6A—C6—H6B	109.5
N1—C4—O1	109.60 (10)	C5—C6—H6C	109.5
N1—C4—C3	113.06 (10)	H6A—C6—H6C	109.5
O1—C4—C3	107.92 (9)	H6B—C6—H6C	109.5
N1—C4—H4	108.7	C11—C10—C9	122.19 (12)
O1—C4—H4	108.7	C11—C10—F1	118.33 (13)
C3—C4—H4	108.7	C9—C10—F1	119.47 (13)
O2—C2—C1	106.93 (10)	C2—C1—H1A	109.5
O2—C2—C3	109.87 (10)	C2—C1—H1B	109.5
C1—C2—C3	112.96 (12)	H1A—C1—H1B	109.5
O2—C2—H2	109.0	C2—C1—H1C	109.5
C1—C2—H2	109.0	H1A—C1—H1C	109.5
C3—C2—H2	109.0	H1B—C1—H1C	109.5
C8—C7—N1	120.04 (11)		
			
C2—O2—C5—O1	61.43 (13)	C4—C3—C2—C1	171.55 (11)
C2—O2—C5—C6	−179.33 (10)	C4—N1—C7—C8	168.84 (12)
C4—O1—C5—O2	−64.23 (12)	C4—N1—C7—C12	−10.76 (19)
C4—O1—C5—C6	176.73 (10)	N1—C7—C8—C9	−177.40 (13)
C7—N1—C4—O1	74.98 (14)	C12—C7—C8—C9	2.2 (2)
C7—N1—C4—C3	−164.58 (11)	C7—C8—C9—C10	−0.1 (2)
C5—O1—C4—N1	−176.11 (9)	C8—C7—C12—C11	−2.58 (19)
C5—O1—C4—C3	60.37 (12)	N1—C7—C12—C11	177.03 (12)
C2—C3—C4—N1	−175.84 (10)	C7—C12—C11—C10	0.8 (2)
C2—C3—C4—O1	−54.45 (12)	C12—C11—C10—C9	1.4 (2)
C5—O2—C2—C1	−178.06 (10)	C12—C11—C10—F1	−178.74 (12)
C5—O2—C2—C3	−55.13 (13)	C8—C9—C10—C11	−1.7 (2)
C4—C3—C2—O2	52.25 (14)	C8—C9—C10—F1	178.39 (12)

### Hydrogen-bond geometry (Å, º)

**Table:**

*D*—H···*A*	*D*—H	H···*A*	*D*···*A*	*D*—H···*A*
N1—H1···F1^i^	0.86	2.48	3.1556 (14)	136

Symmetry code: (i) *x*+1/2, −*y*−1/2, *z*−1/2.
